# Characterization of a New S8 serine Protease from Marine Sedimentary *Photobacterium* sp. A5–7 and the Function of Its Protease-Associated Domain

**DOI:** 10.3389/fmicb.2016.02016

**Published:** 2016-12-22

**Authors:** Hui-Juan Li, Bai-Lu Tang, Xuan Shao, Bai-Xue Liu, Xiao-Yu Zheng, Xiao-Xu Han, Ping-Yi Li, Xi-Ying Zhang, Xiao-Yan Song, Xiu-Lan Chen

**Affiliations:** ^1^State Key Laboratory of Microbial Technology, Marine Biotechnology Research Center, Institute of Marine Science and Technology, Shandong UniversityJinan, China; ^2^College of Chemical and Environmental Engineering, Shandong University of Science and TechnologyQingdao, China

**Keywords:** subtilase, protease-associated domain, marine sediment, collagen-binding, aromatic residues

## Abstract

Bacterial extracellular proteases are important for bacterial nutrition and marine sedimentary organic nitrogen degradation. However, only a few proteases from marine sedimentary bacteria have been characterized. Some subtilases have a protease-associated (PA) domain inserted in the catalytic domain. Although structural analysis and deletion mutation suggests that the PA domain in subtilases is involved in substrate binding, direct evidence to support this function is still absent. Here, a protease, P57, secreted by *Photobacterium* sp. A5-7 isolated from marine sediment was characterized. P57 could hydrolyze casein, gelatin and collagen. It showed the highest activity at 40°C and pH 8.0. P57 is a new subtilase, with 63% sequence identity to the closest characterized protease. Mature P57 contains a catalytic domain and an inserted PA domain. The recombinant PA domain from P57 was shown to have collagen-binding ability, and Phe349 and Tyr432 were revealed to be key residues for collagen binding in the PA domain. This study first shows direct evidence that the PA domain of a subtilase can bind substrate, which provides a better understanding of the function of the PA domain of subtilases and bacterial extracellular proteases from marine sediment.

## Introduction

Organic nitrogen degradation is an important part of marine nitrogen cycle (Aluwihare et al., 2005). Particulate organic nitrogen (PON) that deposits to marine sediments is mainly decomposed by bacterial extracellular proteases, which is generally considered to be the initial and rate-limiting step of nitrogen cycle in marine sediments (Talbot and Bianchi, 1997; Brunnegård et al., 2004). It has been found that protease-producing bacteria and their extracellular proteases are rich and diverse in marine sediments (Olivera et al., 2007; Zhou et al., 2009, 2013). Some proteases from marine sedimentary bacteria have been characterized, most of which are shown to have special properties, such as cold adaptation (Chen et al., 2007; Yan et al., 2009; Kurata et al., 2010; Yang et al., 2013), salt tolerance (Yan et al., 2009), distinct substrate specificity and catalytic mechanism (Ran et al., 2014). Therefore, marine sediments are good resources for exploring novel proteases.

Peptidase family S8, also known as the subtilisin or subtilase family, is the second-largest family of serine proteases (Rawlings et al., 2010). Proteases in this family are all characterized by an Asp/His/Ser catalytic triad and an α/β fold catalytic center containing a seven-stranded parallel β-sheet (Rawlings et al., 2006). Some proteases in the S8 family contain a protease-associated (PA) domain, which is inserted in the catalytic domain. This kind of proteases are reported from both plants and bacteria, such as tomato SBT3 (Ottmann et al., 2009), soybean protease C1 (Tan-Wilson et al., 2012), streptococcal C5a peptidase (Brown et al., 2005; Kagawa et al., 2009), lactococcal cell-envelope protease (Bruinenberg et al., 1994; Sadat-Mekmene et al., 2011), VapT from *Vibrio metschnikovii* RH530 (Kwon et al., 1995), Apa1 from *Pseudoalteromonas* sp. AS-11 (Dong et al., 2001), SapSh from *Shewanella* sp. Ac10 (Kulakova et al., 1999) and AcpII from *Alkalimonas collagenimarina* AC40 (Kurata et al., 2010).

The PA domains in subtilases were reported to have diverse functions. For plant subtilases, the PA domain of tomato SBT3 was suggested to be required for enzyme maturation, secretion, dimerization and activation (Cedzich et al., 2009; Ottmann et al., 2009); homology modeling and molecular simulation indicated that the PA domain of soybean protease C1 was crucial for determining the optimum length of peptide substrate (Tan-Wilson et al., 2012). For bacterial subtilases, deletion experiment indicated that the PA domain of lactococcal cell-envelope protease influenced the enzyme substrate specificity, but was unnecessary for enzyme folding or autoprocessing (Bruinenberg et al., 1994); the PA domains of AcpII from *Alkalimonas collagenimarina* AC40 and of streptococcal C5a peptidase SCPA from *Streptococcus pyogenes* were reported to restrict substrate access to the active site (Kagawa et al., 2009; Kurata et al., 2010). Although structural analyses and deletion experiments suggest that the PA domains in subtilases are involved in substrate binding, there has been no evidence showing that the PA domain of subtilase can bind substrate directly.

In a previous study, 66 protease-producing strains were screened from 6 sediment samples from Jiaozhou Bay, China, in which *Photobacterium* (39.4%), *Bacillus* (25.8%), *Vibrio* (19.7%) and *Shewanella* (7.6%) were the major groups and *Photobacterium* strains were distributed in all sediment samples. Among the strains, *Photobacterium* sp. A5–7 had the highest protease activity, which was isolated from the sediment sample from the A5 station site where the depth, temperature, pH and carbon/nitrogen ratio were 5.9 m, 24.7°C, 8.11 and 7.0, respectively (Zhang et al., 2015). In this study, we aimed to purify and characterize the protease secreted by *Photobacterium* sp. A5–7. The result showed that the protease P57 secreted by *Photobacterium* sp. A5–7 could hydrolyze casein, gelatin and collagen. We then cloned the gene encoding P57. Sequence analysis showed that P57 is a subtilase of the S8 family, containing a PA domain inserted in its catalytic domain. To study its function, the PA domain of P57 was expressed in *Escherichia coli* as a EGFP-fused protein, and its collagen-binding ability was determined by using fluorescent technology. The recombinant PA domain was shown to have collagen-binding ability, suggesting that the PA domain is likely involved in substrate binding during the hydrolysis of insoluble collagen by P57. Site-directed mutations were also performed to analyze the key residues for collagen binding in the PA domain of P57. Our results reveal a new S8 subtilase from a marine sedimentary bacterium and show direct evidence that the PA domain of a subtilase can bind substrate, which shed light on marine bacterial proteases and marine PON degradation.

## Materials and Methods

### Phylogenetic Analysis of Strain A5-7

Genomic DNA of strain A5-7 was extracted using a bacterial genomic DNA isolation kit (BioTeke, China). Using the genomic DNA as template, the 16S rRNA gene of strain A5–7 was amplified by PCR with primers 27F and 1492R (Lane, 1991) and sequenced at Shanghai Biosune Biotechnology Corp. (China). The 16S rRNA gene sequence of strain A5–7, deposited in GenBank database under the accession number JX134463, was compared with those in GenBank and EzTaxon^1^ (Kim et al., 2012) databases using BLASTN (Altschul et al., 1997) to determine the approximate phylogenetic affiliation and select reference sequences of related species for subsequent phylogenetic analysis. The 16S rRNA gene sequence of strain A5–7 was aligned with those of type strains of closely related species using the Clustal W program (Thompson et al., 1994) in the MEGA 5 (Tamura et al., 2011). The alignment obtained was manually trimmed to remove the uneven 5′ and 3′ ends. Phylogenetic trees were constructed using the neighbor-joining (Saitou and Nei, 1987) and maximum-likelihood (Felsenstein, 1981) methods with MEGA 5 (Tamura et al., 2011). Bootstrap analyses (1000 replications) were performed to evaluate tree topologies. Evolutionary distances were calculated using the model of Jukes and Cantor (1969).

### Purification of the Protease P57 Secreted by Strain A5–7

Strain A5–7 was cultivated at 15°C for 72 h in a marine LB medium supplemented with 0.3% (w/v) casein and 0.5% (w/v) gelatin in a rotatory shaker at 180 rpm. The culture was centrifuged at 12,000 *g* and 4°C for 15 min. The cell-free supernatant was concentrated against polyethylene glycol (PEG) 20,000, and then dialyzed against 20 mM phosphate buffer (pH 7.0). The sample was loaded onto a DEAE-Sepharose Fast Flow column (GE Healthcare, USA) equilibrated with the same buffer. Bound proteins were eluted with a linear gradient of 0-1 M NaCl in 20 mM phosphate buffer (pH 7.0). The protease activity of every fraction (4 mL) was measured with casein as substrate. Fractions having protease activity were further subjected to 12.5% SDS-PAGE. Then, fractions having protease activity and displaying a single band in SDS-PAGE gel were collected for further use. The purified protease was named P57.

### Analysis of the N-Terminal Amino Acid Sequence of Protease P57

The purified P57 was transferred from SDS-PAGE gel to a Sequi-Blot polyvinylidene difluoride membrane (PVDF membrane, Bio-Rad, USA). Its N-terminal amino acid sequence was determined by the Edman degradation method with a PROCISE491 sequencer (Applied Biosystems, USA) at Peking University, China. The obtained N-terminal sequence of P57 was aligned with those of proteases in GenBank using BLASTP to determine the protease type.

### Characterization of Protease P57

The activity of P57 was measured as described by Chen et al. (2003) with 2% (w/v) casein as substrate. The optimal temperature of P57 was determined over the range from 0 to 70°C in 20 mM phosphate buffer (pH 7.0). The effect of temperature on protease stability was evaluated by measuring the residual activity at 40°C after P57 was incubated at 30°C, 40°C, or 45°C for different time intervals (5, 10, 15, 20, 30, 40, or 60 min). The optimum pH of P57 was assayed at 40°C in the following buffers (20 mM): Na_2_HPO_4_-citric acid (pH 4.0–6.0), Na_2_HPO_4_-NaH_2_PO_4_ (pH 6.0–8.0), Tris-HCl (pH 8.0–10.0), and Na_2_CO_3_-NaHCO_3_ (pH 9.0–11.0). To evaluate the effect of NaCl on enzyme activity, the assay was carried out at 40°C and pH 8.0 with different salt concentrations from 0 to 3 M. The effects of enzyme inhibitors (PMSF (phenylmethylsulfonyl fluoride), EDTA (ethylene diamine tetraacetic acid), EGTA (ethylene glycol tetraacetic acid), *o*-P (*o*-phenanthroline), and IA (iodoacetic acid)) and metal ions (K^+^, Li^+^, Ba^2+^, Ca^2+^, Co^2+^, Cu^2+^, Mg^2+^, Mn^2+^, Ni^2+^, Sr^2+^, Zn^2+^, and Fe^3+^) on the activity of P57 were evaluated by measuring the enzyme activity at 40°C and pH 8.0 after the enzyme was pre-incubated with each inhibitor or metal ion for 1 h at 4°C.

Substrate specificity of protease P57 was determined by measuring its activities toward casein, gelatin, collagen (Bovine-insoluble type I collagen fiber, Worthington Biochemical Corp., USA), elastin and synthetic peptides. The activities of protease P57 toward collagen and gelatin were measured by the method as described by Worthington Biochemical Corp. (Freehold, 1972). For collagen, the mixture of 1 mL enzyme solution (0.2 mg/mL) and 5 mg collagen was stirred at 37°C for 5 h. One unit is defined as the amount of enzyme that released 1 μmol leucine from collagen in 1 h. For gelatin, the mixture of 100 μL enzyme solution (0.04 mg/mL) and 100 μL of 2% (w/v) gelatin were incubated at 40°C for 10 min. One unit is defined as the amount of enzyme that released 1 μmol leucine from gelatin in 1 min. The activity of protease P57 toward elastin was assayed by the method of Chen et al. (2009). The activities of protease P57 toward synthetic peptides were determined in 20 mM phosphate buffer (pH 8.0) at 40°C according to Peek’s method (Peek et al., 1993). One unit was defined as the amount of enzyme that catalyzed the formation of 1 μmol *p*-nitroaniline in 1 min (Chen et al., 2007). The synthetic peptides used as substrate (0.2%, w/v) were as follows, *N*-succinyl-Ala-Ala-Pro-Leu-*p*-nitroanilide (AAPL), *N*-succinyl-Ala-Ala-Pro-Phe-*p*-nitroanilide (AAPF), *N*-succinyl-Phe-Ala-Ala-Phe-*p*-nitroanilide (FAAF), *N*-succinyl-Ala-Ala-Pro-Arg-*p*-nitroanilide (AAPR), *N*-succinyl-Ala-Ala-Pro-Lys-*p*-nitroanilide (AAPK) and *N*-succinyl-Ala-Ala-Val-Ala-*p*-nitroanilide (AAVA). Protein concentrations were determined by the Bradford method (Bradford, 1976) with bovine serum albumin (BSA) as the standard.

### Gene Cloning and Sequence Analysis of P57

Based on the N-terminal amino acid sequence of P57 and the conserved sequence of the catalytic center of serine proteases (Maciver et al., 1994), two degenerated primers (P57N and RM6) were designed (**Table 1**). With the degenerated primers and the genomic DNA of strain A5–7 as template, a part of the gene encoding P57 was amplified by PCR and sequenced at Shanghai Biosune Biotechnology Corp. (China). By using specific primers and general primers (**Table 1**), the neighboring sequences of the obtained gene fragment were amplified by thermal asymmetric interlaced PCR (TAIL-PCR) (Liu and Whittier, 1995). Through assembly, an entire gene sequence containing an ATG start codon and a TAA stop codon was obtained. Two specific primers (A5–7N and A5–7C) were designed (**Table 1**) according to this ORF, and the full gene encoding P57 was amplified by PCR from the genomic DNA of strain A5–7, and verified by sequencing. The gene sequence of P57 was deposited in GenBank database under the accession number KT923662.

**Table 1:** Primers used in this study.

Gene	Primer type	Sequence
*P57*	Degenerated primers	P57N: 5′-TCCCAAAGCCTTCCNTGGGGNCA-3′
		RM6: 5′-GGNACNTCNATGGCNACNCC-3′
	5′-region specific primers	U1: 5′-TTACCGCTGAGATCGTTGTGTG-3′
		U2: 5′-CGTTGTGTGCAAGATCGTAGCCTG-3′
		U3: 5′-CAATGATACACACGGTGCGGTTAC-3′
	General primer	ADn: 5′-AAKYRTATG-3′
	3′-region specific primers	D1: 5′-GCAGCTACAACCTTGTTTCGG-3′
		D2: 5′-TGTTTCGGTATCTGTCGATCGCAC-3′
		D3: 5′-GCACCCTTGGTTTGGAACTGGC-3′
	General primer	ADc: 5′-GCAGCGTTA-3′
	Specific primers	A5-7N: 5′-TTCCATGAACAAGAACTATAAC-3′
		A5-7C: 5’-TTTAAAGTTGATACGCCAGC-3’
*PA-EGFP*	Overlapping primers	PA-N: 5′-CGCCATATGGATATTACCTTAGCAGGACAG-3′(NdeI)
		PA-GFP1: 5′-CTCCTCGCCCTTGCTCAC ACTATCGACAGTTATTG-3′
		PA-GFP2: 5′-AATAACTGTCGATAGTGTGAGCAAGGGCGAGGAG-3′
		PA-GFP(C): 5′-CGCGCTCGAGCTTGTACAGCTCGTCCATG-3′(XhoI)


*Note, the underlined sequences indicate restricted enzyme sites.*

The domain architectures of P57 were analyzed by the Conserved Domain Database (CDD) of NCBI^2^ (Marchlerbauer et al., 2007). Homologous sequences to P57 were searched using the BLAST against NCBI nr database and the MEROPS database^3^ (Rawlings et al., 2006) with default parameters. The representative homologs to P57 were aligned by using DNAMAN software.

### Expression and Purification of the PA Domain and Its Site-directed Mutants

The DNA fragment encoding the PA domain of protease P57 with an overlapping sequence of EGFP was amplified by PCR using the genomic DNA of strain A5–7 as the template and two primers (PA-N, PA-GFP1) (**Table 1**). The DNA fragment encoding EGFP with the same overlapping sequence was also amplified by PCR using the vector p*EGFP-N1* (Clontech, USA) as the template and the primers PA-GFP2 and PA-GFP(C) (**Table 1**). The two fragments were concatenated by the overlapping extension PCR (Liu and Whittier, 1995). The chimeric gene was sub-cloned into the NdeI-XhoI site of pET-22b (+) to construct the expression vector pET-22b-*PA-EGFP*, which was then transformed into *E. coli* BL21 (DE3). The recombinant PA-EGFP was expressed as a C-terminal His_6_-tagged protein. The transformant cells were cultivated in a 100 mL LB medium supplemented with 100 μg/mg ampicillin at 37°C and 180 rpm, until the absorbance at 600 nm reached 1.5. Then, isopropyl β-D-thiogalactopyranoside (IPTG) was added to a final concentration of 0.1 mM. After induction for 24 h at 15°C and 150 rpm, cells were harvested by centrifugation. Cell pellets were suspended in a 35 mL lysis buffer (50 mM Tris-HCl pH 9.0) and disrupted by sonication. After centrifugation at 4°C and 12,000 *g* for 30 min, the supernatant was collected and the recombinant PA-EGFP protein was purified with a HisBind metal chelating column.

Alignment of the PA domains from P57 and other reported S8 subtilases were performed by using Clustal W. The conserved aromatic residues and positively charged residues were chosen for mutagensis. Site-directed mutagenesis on the PA domain was carried out by overlapping extension PCR (An et al., 2005) using the vector pET-22b-*PA-EGFP* as template. Mutated sites were introduced by the primers with single-point mutations. The mutated genes were sub-cloned into pET-22b (+) and transformed into *E. coli* BL21 (DE3). All mutations were confirmed by enzyme digestion and nucleotide sequencing. The expression and purification of the mutants were performed under the same conditions as those of PA-EGFP.

### Analysis of the Binding Ability of Wild P57, the PA Domain and Its Mutants to Bovine-Insoluble Type I Collagen Fiber

To analyze the binding ability of the PA domain and its mutants toward collagen, 5 mg collagen fibers were mixed with 500 μL PA-EGFP, EGFP or the mutants (0.5 mg/mL) in 50 mM Tris-HCl buffer (pH 8.0). The mixture was incubated at 37°C for 2 h, and then centrifuged at 12,000 *g* and 4°C for 10 min. The free fluorescence intensity in the supernatant before and after incubation was measured on a FP-6500 spectrofluorometer (Jasco, Japan). Fraction of fusion protein bound (%) = (B-A)/B*100%, B and A represent the free fluorescence intensity in the solution before and after a fusion protein binds to collagen, respectively.

The collagen-binding ability of wild P57 was assayed as described by Itoi et al. (2006). Purified P57 (0.2 mg/mL, 0.5 mL) in 20 mM Na_2_HPO_4_-NaH_2_PO_4_ (pH 7.0) containing 10 mM Fe^3+^ was incubated at 4°C for 1 h to inhibit the enzyme activity. Then collagen fibers (1, 5, or 10 mg) was added and the mixture was incubated at 37°C for 2 h with stirring. After incubation, the mixture was centrifuged for 10 min at 12,000 *g* and 4°C. The supernatant was analyzed by 12.5% SDS-PAGE. BSA as a negative control was treated as P57.

### Analysis of the PA Domain and Its Mutants by Circular Dichroism

Circular dichroism (CD) spectra of the purified PA-EGFP and its mutants F349A-EGFP and Y432A-EGFP in 50 mM Tris-HCl buffer (0.1 mg/mL) were measured on a Jasco J810 spectropolarimeter (Japan) according to the method described by Zhao et al. (2008).

## Results

### Phylogenetic Analysis of Strain A5–7

**FIGURE 1:**
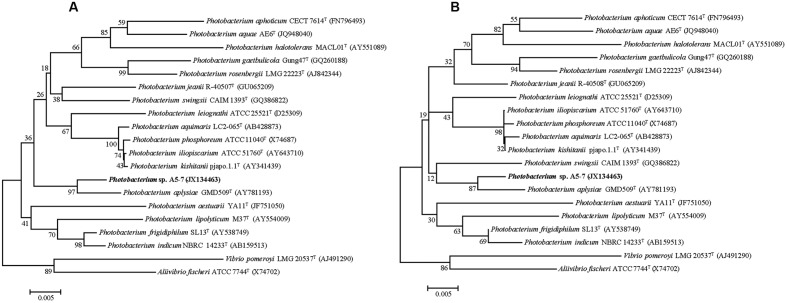
**Neighbor-joining** **(A)** and Maximum-likelihood **(B)** phylogenetic trees based on the 16S rRNA gene sequences of strain A5–7 and type strains of species from the *Photobacterium* genus and other related genera. Bootstrap percentages from 1,000 replicates are indicated at nodes. Bar, 0.005 substitutions per nucleotide position.

Strain A5–7 was isolated from marine sediment collected from A5 station in Jiaozhou Bay, China, and the nearly complete 16S rRNA gene sequence (1536 bp) of the strain was determined. Sequence comparison showed that strain A5–7 shared the highest 16S rRNA gene sequence identities with known *Photobacterium* species, suggesting an affiliation with the genus *Photobacterium*. In the neighbor-joining and maximum-likelihood trees (**Figures 1A,B**) based on the 16S rRNA gene sequences, strain A5–7 fell within the clade of the genus *Photobacterium* and formed a distinct intra-branch with type strain of *Photobacterium aplysiae* supported by high bootstrap values (>85%), indicating its close phylogenetic relationship to the latter. However, considering that strain A5–7 has only 96.8% 16S rRNA gene sequence identity to the type strain of *P. aplysiae*, strain A5–7 may represent a potential new species of the genus *Photobacterium*, which merits further study.

### Purification and Characterization of Protease P57 Secreted by *Photobacterium* sp. A5–7

**FIGURE 2:**
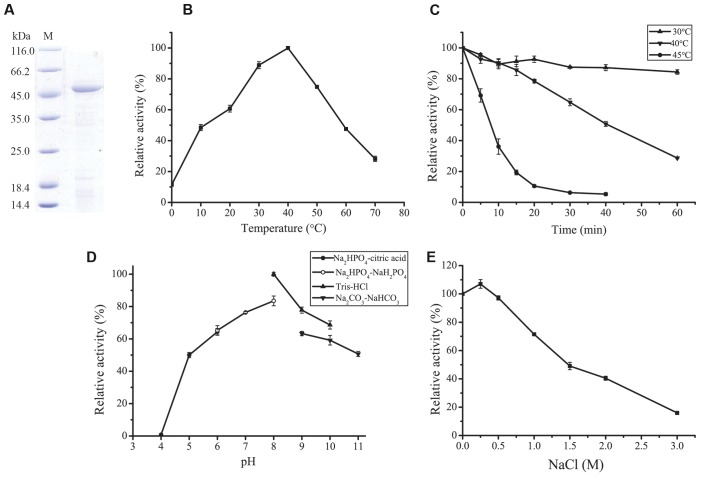
**Purification and characterization of P57.** **(A)** SDS-PAGE analysis of the purified P57. M, protein molecular mass marker; Lane 1, the purified P57. **(B)** Effect of temperature on P57 activity. Enzyme activity was measured at 0–70°C in 20 mM phosphate buffer (pH 7.0). **(C)** Thermostability of P57. The enzyme was incubated at 30°C, 40°C, or 45°C for different time periods, and then residual activity was measured at 40°C. **(D)** Effect of pH on the activity of P57. Enzyme activity was measured at 40°C in different pH buffers (20 mM): Na_2_HPO_4_-citric acid (pH 4.0–6.0), Na_2_HPO_4_-NaH_2_PO_4_ (pH 6.0–8.0), Tris-HCl (pH 8.0–10.0), and Na_2_CO_3_-NaHCO_3_ (pH 9.0–11.0). **(E)** Effect of NaCl on the activity of P57. Enzyme activity was measured at 40°C in different concentrations of NaCl. The graph shows data from triplicate experiments (mean ± SD).

A protease was purified from the culture of strain A5–7 by anion exchange chromatography with a yield of 15.9%. SDS-PAGE analysis showed that the purified protease had high purity, with an apparent molecular mass of approximately 45 kDa (**Figure 2A**). This protease was named P57 in this study.

With casein as substrate, the optimum temperature of P57 was 40°C, and 10% of the maximum activity was retained at 0°C (**Figure 2B**). P57 was stable at 30°C for at least 1 h, but unstable at temperatures higher than 30°C. The half time of its activity at 40°C and 45°C was 40 min and 7 min, respectively (**Figure 2C**). P57 was active in a wide range of pH from 5.0 to 11.0, with the maximum activity at pH 8.0 (**Figure 2D**). In the buffers containing 0–3 M NaCl, the activity of P57 peaked at 0.25 M NaCl, then declined with the increase of NaCl concentration, but still retained 50% of the maximum activity at 1.5 M NaCl (**Figure 2E**).

Effects of metal ions and inhibitors on P57 activity were shown in **Table 2**. P57 activity was not affected by Li^+^, K^+^, Ba^2+^, Co^2+^, or Sr^2+^. At the final concentration of 8 mM, Cu^2+^, Zn^2+^, and Ni^2+^ obviously inhibited P57 activity, and Fe^3+^ severely inhibited P57 activity by 96.4%. While Ca^2+^ and Mg^2+^ slightly activated the enzyme activity, Mn^2+^ significantly increased P57 activity to 194.1%, showing an activating effect on P57 activity. In addition, P57 activity was strongly inhibited by PMSF, a serine protease inhibitor, indicating that P57 is likely a serine protease. The activity of P57 was also inhibited by metal chelators EDTA and EGTA, but not affected by *o*-P or IA.

**Table 2:** Effects of metal ions and inhibitors on the activity of protease P57.

Metal ions	Relative activity (%)		Metal ions	Relative activity (%)		Inhibitor	Residual activity (%)
					
	2 mM*^b^*	8 mM*^b^*		2 mM*^b^*	8 mM*^b^*		
Control*^a^*	100	100				Control*^a^*	100
K^+^	92.8 ± 1.5	95.4 ± 3.2	Mg^2+^	98.4 ± 3.5	107.6 ± 0.4*	PMSF (1 mM*^c^*)	2.8 ± 0.1
Li^+^	99.4 ± 1.4	87.7 ± 3.0	Mn^2+^	142.1 ± 5.1*	194.1 ± 2.1**	EDTA (1 mM*^c^*)	11.6 ± 1.8
Ba^2+^	101.0 ± 2.0	97.5 ± 1.3	Ni^2+^	80.6 ± 2.7*	76.1 ± 1.5**	EGTA (1 mM*^c^*)	17.9 ± 1.3
Ca^2+^	92.2 ± 1.1	110.4 ± 2.1*	Sr^2+^	97.8 ± 1.6	102.3 ± 1.9	*o*-P (5 mM*^c^*)	79.4 ± 1.8
Co^2+^	102.8 ± 1.3	99.0 ± 3.3	Zn^2+^	102.3 ± 2.2	67.1 ± 1.3**	IA (5 mM*^c^*)	82.7 ± 2.5
Cu^2+^	103.6 ± 1.8	62.2 ± 2.1**	Fe^3+^	99.8 ± 2.9	3.6 ± 0.3**		


*^*a*^The activity of P57 without any metal ion or inhibitor was taken as control. ^*b*^The final concentration of metal ion in the reaction mixture. ^*c*^The final concentration of inhibitor in the reaction mixture.*

*^∗^, ^∗∗^ stand for different significance at *p* < 0.05 and *p* < 0.01, respectively. The data shown are the means of three repeats with standard deviations. PMSF, phenylmethylsulfonyl fluoride; EDTA, ethylene diamine tetraacetic acid; EGTA, ethylene glycol tetraacetic acid; *o*-P, *o*-phenanthroline; IA, iodoacetic acid.*

Substrate specificity analysis showed that P57 could hydrolyze casein, gelatin and collagen, with the highest activity toward gelatin, the degenerated form of collagen (**Table 3**). P57 showed no activity toward elastin. Among the synthetic peptides, P57 had higher activities toward AAPL and AAPF and lower activities toward FAAF, AAPR, AAPK and AAVA (**Table 3**).

**Table 3:** Substrate specificity of P57 toward various proteins and synthetic peptides^a^.

Substrate	Specific activity (U/mg)	Substrate	Specific activity (U/mg)
Casein	2,460.42 ± 62.68	AAPL	3.84 ± 0.06
Gelatin	15,878.57 ± 202.51	AAPF	3.29 ± 0.15
Collagen	298.13 ± 58.31	FAAF	1.96 ± 0.05
Elastin	–	AAPR	1.75 ± 0.02
		AAPK	0.75 ± 0.01
		AAVA	0.70 ± 0.02


**^a^*The data shown are the means of three repeats with standard deviations. –, No detectable activity. AAPL, *N*-succinyl-Ala-Ala-Pro-Leu-*p*-nitroanilide; AAPF, *N*-succinyl-Ala-Ala-Pro-Phe-*p*-nitroanilide; FAAF, *N*-succinyl-Phe-Ala-Ala-Phe-*p*-nitroanilide; AAPR, *N*-succinyl-Ala-Ala-Pro-Arg-*p*-nitroanilide; AAPK, *N*-succinyl-Ala-Ala-Pro-Lys-*p*-nitroanilide; AAVA, *N*-succinyl-Ala-Ala-Val-Ala-*p*-nitroanilide.*

### Gene Cloning and Sequence Analysis of P57

The N-terminal sequence of mature P57 was determined by protein sequencing to be Ser-Gln-Ser-Leu-Pro-Trp-Gly-Gln-Thr-Phe-Val-Gly-Ala-Thr-Leu, which shows identities to some serine proteases of the S8 family, such as SapSh (66.7%) from *Shewanella* sp. Ac10 (Kulakova et al., 1999), Apa1 (66.7%) from *Pseudoalteromonas* sp. AS-11 (Dong et al., 2001), VapT (40%) from *V. metschnikovii* RH530 (Kwon et al., 1995) and AcpII (40%) from *A. collagenimarina* AC40 (Kurata et al., 2010). This suggests that P57 is a serine protease of the S8 family. Based on the N-terminal amino acid sequence of P57 and the highly conserved sequence of the catalytic domains of the S8 serine proteases, the gene encoding protease P57 was cloned from the genomic DNA of strain A5–7 by a combination of PCR and TAIL-PCR.

**FIGURE 3:**
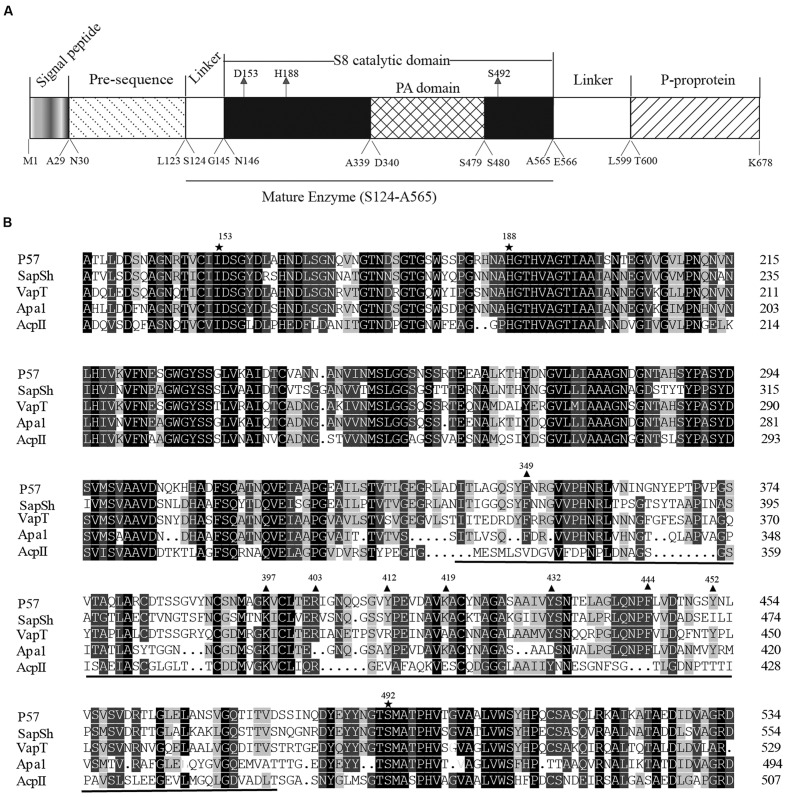
**Sequence analysis of protease P57.** **(A)** The diagram of conserved domains of P57 based on BLAST analysis against Conserved Domain Database. **(B)** Sequence alignment of the catalytic domains of P57 and other reported S8 subtilases with a PA domain. Catalytic residues are indicated with “
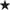
”, and the PA domain is underlined. Identical residues are shaded in black. Conserved amino acid residues indicated with “
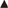
” are the residues chosen for site-directed mutations to determine the key residues for collagen binding in the PA domain. P57, from *Photobacterium*. sp. A5–7 in this study (KT923662); SapSh, from *Shewanella* sp. Ac10^T^ (AAC04871) (Kulakova et al., 1999); VapT, from *Vibrio metschnikovii* strain RH530^T^ (CAA82213) (Kwon et al., 1995); Apa1, from *Pseudoalteromonas* sp. AS-11^T^ (AB120714) (Dong et al., 2001); AcpII, from *Alkalimonas collagenimarina* AC40^T^ (BAI50017) (Kurata et al., 2010).

The ORF of *P57* is 2,037 bp in length, including an ATG start codon and a TAA stop codon. It encodes a protein of 678 amino acid residues with a calculated molecular weight of 71,788 Da. According to BLAST analysis against CDD database (Marchlerbauer et al., 2007), the precursor of P57 contains an N-terminal pre-sequence (Met1-Leu123), an S8 catalytic domain (Asn146-Ala565), a PA domain (Asp340-Ser479) inserted in the catalytic domain, a linker (Glu566-Leu599) and a C-terminal P-proprotein (Thr600-Lys678) (**Figure 3A**). SignalP 3.0 (Bendtsen et al., 2004) prediction suggested that the pre-sequence of P57 contains an N-terminal signal peptide sequence (Met1-Ala29). According to the result of N-terminal sequencing, the first N-terminal residue of mature P57 is Ser124. The molecular mass of mature P57 was determined to be 46,016 Da by MALDI-TOF mass spectrometry. Thus, based on the sequence and the molecular mass of P57, it was predicted that mature P57 contains 442 residues from Ser124 to Ala565. Therefore, both the N-terminal pre-sequence and the C-terminal *P*-proprotein are cleaved off during enzyme maturation, and mature P57 only contains the catalytic domain and the inserted PA domain (**Figure 3A**). Multiple sequence alignment suggests that the catalytic triad of P57 is composed of Asp153, His188 and Ser492 (**Figure 3B**). The highest sequence identity of P57 with reported S8 serine proteases is 63% according to BLASTP analysis against NCBI non-redundant protein database.

### Collagen Binding Ability of the PA Domain

**FIGURE 4:**
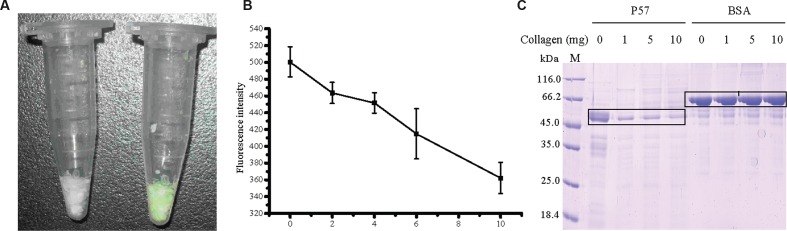
**Analysis of the collagen-binding ability of the recombinant PA domain.** **(A)** The binding ability of EGFP (left tube) and PA-EGFP (right tube) on bovine-insoluble type I collagen. After the mixture of EGFP or PA-EGFP with 5 mg collagen was incubated at 37°C for 2 h with agitating and subsequently centrifuged, the precipitated collagen fibers were washed two times with distilled water. **(B)** Fluorescence analysis of the collagen-binding ability of the PA domain. The fusion protein PA-EGFP was incubated with various amounts of collagen at 37°C for 2 h. After centrifugation, the fluorescence intensities of the supernatants were determined at 510 nm. PA-EGFP incubated with no collagen at the same conditions was served as control. The graph shows data from triplicate experiments (mean ± SD). **(C)** SDS-PAGE analysis of the binding ability of P57 to bovine insoluble type I collagen in the presence of 10 mM Fe^3+^. P57 in 20 mM Na_2_HPO_4_-NaH_2_PO_4_ (pH 7.0) containing 10 mM Fe^3+^ was incubated at 4°C for 1 h to inhibit the enzyme activity, and then was mixed with 0, 1, 5, or 10 mg collagen. The mixtures were incubated at 37°C for 2 h, and the unbound P57 fractions in the mixtures were analyzed by 12.5% SDS-PAGE. BSA was used as a negative control. M, protein molecular mass marker.

To study the function of the PA domain in P57 catalysis, the PA domain fused with EGFP (PA-EGFP) was expressed in *E. coli* BL21 (DE3), and purified by HisBind metal chelating column with a yield of 51.7%. Because P57 could hydrolyze insoluble collagen, we investigated the binding ability of the recombinant PA domain to insoluble collagen by fluorescence analysis. After PA-EGFP was mixed with insoluble collagen fibers for 2 h at 37°C, the precipitated collagen fibers displayed a bright green color, whereas the collagen fibers mixed with EGFP still displayed its own white color (**Figure 4A**). This result suggests that the PA domain of P57 has collagen-binding ability. To further confirm this, we quantified the fluorescence intensity changes of the supernatant with different amounts of collagen in the mixture. As shown in **Figure 4B**, the fluorescence intensity in the supernatant decreased from 500 to 360 with the increase of collagen amount from 0 to 10 mg in the mixture, which indicated that the amount of PA-EGFP bound to collagen fibers increased with the increase of collagen amount in the mixture, thereby leading to a successive decrease of PA-EGFP amount in the solution. Taken together, these results indicate that the PA domain of P57 has collagen-binding ability, implying that the PA domain in P57 may be involved in substrate binding in the catalysis of P57 toward collagen.

In addition, to estimate the contribution of the PA domain to the interaction of P57 with collagen, the collagen-binding ability of P57 was assayed after the enzyme activity was inhibited by 10 mM Fe^3+^. As shown in **Figure 4C**, after P57 was incubated with collagen at 37°C for 2 h in the presence of 10 mM Fe^3+^, the amount of P57 protein in the supernatant significantly decreased, whereas the amount of BSA in the supernatant showed little change after incubation with collagen (**Figure 4C**). This result indicated that P57 can bind to collagen at 37°C. The PA domains probably play an important role in the interaction of P57 with collagen because it has collagen-binding ability (**Figures 4A,B**).

### Key Residues for Collagen Binding in the PA Domain

**FIGURE 5:**
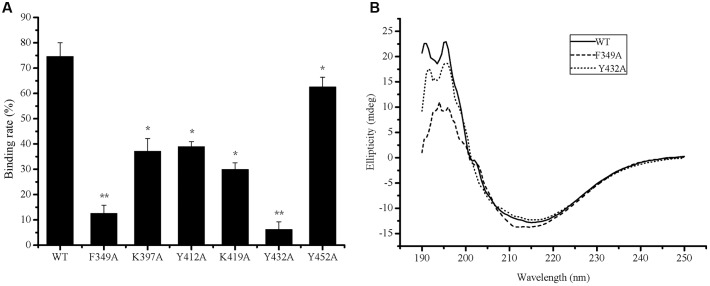
**Collagen-binding affinity and circular dichroism spectra of PA-EGFP and its mutants.** **(A)** The collagen-binding affinity of PA-EGFP (WT) and its mutants. The graph shows data from triplicate experiments (mean ± SD). ^∗^, ^∗∗^ stand for different significance at *p* < 0.05 and *p* < 0.01, respectively. **(B)** Circular dichroism spectra of PA-EGFP (WT) and its mutants F349A-EGFP (F349A) and Y432A-EGFP (Y432A).

To determine the key amino acid residues in the PA domain of P57 for collagen binding, site-directed mutagenesis was performed. It has been reported that aromatic residues and charged residues usually play a key role in the binding of binding domains to insoluble substrates (Bhaskaran et al., 2008; Philominathan et al., 2009). Based on sequence alignment of the PA domains from P57 and other reported proteases (**Figure 3B**), conserved aromatic residues (Phe349, Tyr412, Tyr432, Phe444 and Tyr452) and conserved positively charged residues (Lys397, Arg403 and Lys419) in the PA domain of P57 were mutated to Ala, and all the mutants were expressed as EGFP-fused proteins. The mutants R403A-EGFP and F444A-EGFP could not be expressed as soluble proteins, probably because these two residues are important for the correct folding of the proteins. The binding abilities of the mutants to collagen were measured and compared with that of PA-EGFP. While the collagen-binding abilities of mutants K397A-EGFP, Y412A-EGFP, K419A-EGFP and Y452A-EGFP were partly reduced, the collagen-binding abilities of mutants F349A-EGFP and Y432A-EGFP were severely destroyed, implying that Phe349 and Tyr432 may be essential for the PA domain to bind collagen (**Figure 5A**). In addition, CD spectra of PA-EGFP, F349A-EGFP and Y432A-EGFP were collected to analyze the structural changes that residue substitution mutation may cause in the mutants. There were few differences in the spectra of PA-EGFP, F349A-EGFP and Y432A-EGFP (**Figure 5B**), suggesting that the loss of the collagen-binding ability of F349A-EGFP and Y432A-EGFP should be caused by amino acid substitution. Altogether, these data indicate that Phe349 and Tyr432 are likely two key residues for collagen binding in the PA domain of P57. The data also suggest that hydrophobic interactions likely play an important role in the binding of the PA domain to collagen because Phe349 and Tyr432 are both aromatic amino acids.

## Discussion

In this study, the extracellular protease P57 from *Photobacterium* sp. A5–7 isolated from the sea sediment of Jiaozhou Bay in China was characterized. P57 is a serine protease of the S8 family. Among reported S8 serine proteases, P57 has the highest identity (63%) to VapT from *V. metschnikovii* RH530 (Kwon et al., 1995), which suggests that P57 is a new member of the S8 family. Although the precursor of P57 contains an N-terminal pre-sequence, an S8 catalytic domain, a PA domain and a C-terminal *P*-proprotein domain, mature P57 only contains the catalytic domain and the PA domain that is inserted in the catalytic domain. The N-terminal pre-sequence, which is required for protease folding and secretion (Chen and Inouye, 2008), and the C-terminal *P*-proprotein domain, which is also important in enzyme secretion through the cell membrane (Kurata et al., 2007), are all cleaved off during P57 maturation.

Our results show that P57 is a subtilase with an inserted PA domain in its catalytic domain. Some subtilases with an inserted PA domain in the catalytic domain have been reported from plants and bacteria, and structural and deletion mutational analyses have suggested that the PA domains in some subtilases are involved in substrate binding. For example, the crystal structures of C5a peptidases from *S. agalactiae* and *S. pyogenes* show that the PA domain is located near the active-site cleft of the catalytic domain (Brown et al., 2005; Kagawa et al., 2009). Three out of four residues that form the S4 subsite belong to the PA domain in soybean protease C1 (Tan-Wilson et al., 2012). Cleavage specificity toward natural casein substrate was changed in PrtP from *Lactococcus lactis* without the PA domain (Bruinenberg et al., 1994). The activity of AcpII-ΔPA toward gelatin, casein and collagen was remarkably increased (Kurata et al., 2010). Despite these structural and deletion mutational analyses, there is no direct evidence that the PA domain from a subtilase has substrate-binding ability. In this study, the results showed that the PA domain from the subtilase P57 had collagen-binding ability, and that residues Phe349 and Tyr432 are two key residues responsible for collagen binding in the PA domain, showing direct evidence that the PA domain of a subtilase has substrate-binding ability. These results also suggest that the function of the PA domain in P57 is most likely to participate in the binding of insoluble substrate such as collagen during P57 catalysis.

While most subtilases have no collagenolytic activity, some subtilases have been reported to be serine collagenolytic proteases that can degrade insoluble collagen, such as MCP-01 from *Pseudoalteromonas* sp. SM9913 (Zhao et al., 2008; Ran et al., 2013), the thermostable protease from *Geobacillus collagenovorans* MO-1 (Okamoto et al., 2001), AcpII from *Alkalimonas collagenimarina* AC40 (Kurata et al., 2010), and myroicolsin from *Myroides profundi* D25 (Ran et al., 2014). P57 has collagenolytic activity and its PA domain has collagen-binding ability, indicating that P57 is an S8 serine collagenolytic protease. Subtilases, such as SapSh (Kulakova et al., 1999), VapT (Kwon et al., 1995) and AcpII (Kurata et al., 2010), usually have activity toward synthetic peptides AAPL and AAPF. Consistent with this, P57 has obvious activity toward these two peptides. Metal ions have different effects on the activity of different enzymes. It was reported that Ca^2+^ could increase the enzyme activity or the thermal stability of some subtilases (Kurata et al., 2007, 2010; Zhao et al., 2008; Ran et al., 2014). However, Ca^2+^ only had a little effect on P57 activity. Previous reports showed that Mn^2+^ did not affect the enzyme activity of subtilase significantly (Kurata et al., 2007) or slightly inhibited the enzyme activity of subtilase (Zhao et al., 2008; Ran et al., 2014). Our result showed that 8 mM Mn^2+^ significantly increased P57 activity. EDTA and EGTA are ion chelators, which usually inactivate subtilase that contains metal ion and/or lower their stability by depriving the metal ion (Kurata et al., 2010). The activity of P57 was significantly reduced by EDTA and EGTA, implying that P57 may contain metal ion.

Extracellular proteases from marine sedimentary bacteria usually have some environment-adapted characters, including cold-adaptation, salt tolerance/activation, and an optimal pH equal or near that of seawater, such as proteases MCP-01 and MCP-03 from *Pseudoalteromonas* sp. SM9913 (Chen et al., 2003; Zhao et al., 2008; Yan et al., 2009) and pseudoalterin from *Pseudoalteromonas* sp. CF6-2 (Zhao et al., 2012). Consistent with this, P57 also shows some characters adapted to marine sediment environment. *Photobacterium* sp. A5–7 was isolated from a marine sediment sample from the A5 station site in Jiaozhou Bay, China, where the depth, temperature, pH and C/N ration were 5.9 m, 24.7°C, 8.11 and 7.0, respectively (Zhang et al., 2015). P57 had low optimal temperature (40°C), and low thermostability at moderate temperatures (unstable at temperatures higher than 30°C), and displayed high activity at alkaline pH (pH 7.0–9.0) with the optimum pH of 8.0. P57 showed the highest activity at 0.25 M NaCl, and retained 50% of the maximum activity at 1.5 M NaCl. These characters reflect the adaptation of P57 to the marine sedimentary environment. Extracellular proteases of marine sedimentary bacteria play important roles in PON degradation and nitrogen cycling in marine sediments. As a bacterial extracellular protease from marine sediment can degrade proteins and peptides, P57 should be actively involved in sedimentary PON degradation.

In summary, protease P57 secreted by marine sedimentary *Photobacterium* sp. A5–7 was characterized in this study. P57 is a new S8 subtilase that can degrade casein, collagen and gelatin. P57 has a PA domain inserted in its catalytic domain. The PA domain from P57 has collagen-binding ability, which represents the first direct evidence that the PA domain of a subtilase has substrate-binding ability. Phe349 and Tyr432 were further revealed to be key residues responsible for collagen binding in the PA domain of P57 by site-directed mutational analysis. Our results provide more insight into the function of PA domain of subtilases and shed light on marine sedimentary bacterial proteases and PON degradation.

## Author Contributions

H-JL, B-LT, and X-XH performed the biochemical experiments. XS, B-XL, and X-YZ helped in protein purification. X-LC designed and directed the research. H-JL and X-LC wrote the manuscript. P-YL and X-YZ helped in data analysis and manuscript editing.

## Conflict of Interest Statement

The authors declare that the research was conducted in the absence of any commercial or financial relationships that could be construed as a potential conflict of interest.

## Footnotes

1 http://eztaxon-e.ezbiocloud.net/

2 http://www.ncbi.nlm.nih.gov/Structure/cdd

3 http://www.merops.ac.uk
